# The Length and Distribution of Plasma Cell-Free DNA Fragments in Stroke Patients

**DOI:** 10.1155/2020/9054196

**Published:** 2020-01-30

**Authors:** Xiaofang Cui, Shiyi Du, Houlin Liu, Ju Liu, Qingjian Wu, Qing Huo, Yanwei Qi, Xiao Qin, Yan Yang, Weiyang Li

**Affiliations:** ^1^Jining Medical University, Jining, Shandong 272067, China; ^2^Shandong Key Laboratory of Behavioral Medicine, School of Mental Health, Jining Medical University, Jining, Shandong 272067, China; ^3^Guangzhou Root Path Genomics Inc., Guangzhou 510006, China; ^4^Jining No. 1 People's Hospital, Jining, Shandong 272011, China; ^5^Southern University of Science and Technology, Shenzhen 518055, China; ^6^Shenzhen Research Institute of City University of Hong Kong, Shenzhen 518057, China; ^7^Collaborative Innovation Center for Birth Defect Research and Transformation of Shandong Province, Jining Medical University, Jining, Shandong 272067, China

## Abstract

A number of studies have shown that plasma cell-free DNA is closely related to the risk of stroke, but the fragmentation status of plasma cell-free DNA and its clinical application value in ischemic stroke are still unclear. In this study, 48 patients with new ischemic stroke and 20 healthy subjects were enrolled. The second-generation high-throughput sequencing technique was used to study the plasma cell-free fragment length and regional distribution of the subjects. As noted in our results, the ratio of plasma cell-free DNA fragments in the disease group was significantly greater than that of the healthy group in the 300–400 bp range; conversely for fragments at the 75–250 bp range, the ratio of plasma cell-free DNA fragments in the patient group was apparently lower than that of the healthy group. In-depth analysis of the proportion of fragments distributed on each component of the genome was carried out. Our results recorded that the plasma cell-free DNA fragments in the disease group were inclined to the EXON, CpG islands, and ALU regions in contrast to that of the healthy group. In particular, fragments within the 300–400 bp range of the disease group were enrichment in the regions of EXON, INTRON, INTERGENIC, LINE, Fragile, ALU, and CpG islands. In summary, our findings suggested that the intracellular DNA degradation profiles could be applied to distinguish the stroke group and the healthy group, which provided a theoretical basis for the clinical diagnosis and prognosis of stroke by profiling the characteristic of plasma cell-free DNA fragments.

## 1. Introduction

Stroke is an acute cerebrovascular disease characterized by focal neurological deficits. In general, stroke could be subdivided into ischemic stroke (IS) (cerebral infarction) and hemorrhagic stroke (Cerebral hemorrhage). Ischemic stroke accounts for about 60%–80% of stroke cases [1], and the number of incidences continue to raise each year. Ischemic stroke is characterised by high morbidity and mortality. Over recent years, a number of potential biochemical markers associated with stroke had been reported [2]. Yet still, the current clinical practice lacked a rapid, reliable, sensitive, and specific serological test or indicator to assess risks of patients developing ischemic stroke. Therefore, the exploration and development of new serological indicators for the risk evaluation of ischemic stroke will have important significance for the cure rate and reduce the stroke disability and patient mortality.

Plasma cell-free DNA (cf-DNA) refers to the content of extracellular DNA present in human plasma. During cell death, chromosomal DNA is no longer encapsulated in the membrane, but is fragmented. The nucleosome assembly state enters the bloodstream to form plasma cell-free DNA [3]. As a marker of cell death, its monitoring is relatively simple, and the trauma is small when taking materials. The attempts to use cell-free DNA as a diagnostic biomarker have gained tremendous popularity recently, providing new approaches to meet the diagnostic and prognostic demands for various diseases [4, 5]. One of the most noted progresses in the field is the development of noninvasive prenatal diagnosis, helping to reduce the fetal chromosomal abnormal disease (e.g., 21 trisomy) and having now been widely applied in clinical practice [6–8], with an impressive 99% accuracy. Moreover, fetus-derived DNA in the plasma of pregnant women is shorter than maternal DNA [9, 10]. In addition, cell-free DNA derived from tumor cells is shorter than that from nonmalignant cells in the plasma of cancer patients, and tissue necrosis may generate longer DNA fragments [11]. Therefore, size differences might be used for developing size-based diagnostics [12–15].

The correlation report of plasma cell-free DNA and stroke could be tracing back in 2003. Rainer et al. recorded that the cell-free DNA levels in the stroke patients were positively correlated with prognostic mortality [16], suggesting that the quantification of plasma cell-free DNA could enable the prediction of stroke incidence and mortality rate. In addition, several other studies revealed that there were different characteristics of the length of plasma cell-free DNA fragments and genomic regional distribution in different diseases [12, 17, 18]. Altogether, the studies on the fragmentation regularity of plasma cell-free DNA suggested that it could have a strong connection with different diseases, which further implicated potentials in clinical applications. However, the fragmentation state of plasma cell-free DNA in stroke patients and its association with clinical studies remain unclear.

In this study, 20 healthy individuals and 48 patients of ischemic stroke were enrolled. Deploying a second-generation high-throughput sequencing platform, our team had profiled the length and genomic regional distribution of cell-free DNA fragments in the healthy individuals and the ischemic stroke patient. As revealed in our results that the plasma cell-free DNA fragments of 300–400 bp range were highly abundant in the ischemic stroke patients, but not in the healthy individuals; additionally, the cell-free DNA fragmentation length showed certain uniformity. In summary, this research had compared the length and genomic regional distribution of plasma cell-free DNA fragments in the healthy group and the stroke group and tried to identify the characteristic of cell-free DNA in the stroke patients, which could help to generate clinical values for assessing stroke disease status in the near future.

## 2. Methods and Materials

In brief, the entire blood collection procedures were completed within 8 h, and the plasma samples were extracted from whole blood after two rounds of centrifugation. All procedures performed in studies involving human participants were in accordance with the ethical standards of the institutional research committee and with the 1964 Helsinki Declaration and its later amendments or comparable ethical standards. The study had been approved by the Ethics Review Committee in Jining Medical University. The cell-free DNA of plasma samples (healthy = 20 and IS = 48) were subsequently subjected to library construction and sequencing (paired-end 100 bp) by the BGISEQ-500 sequencer according to the BGISEQ-500 protocol. The BWA algorithm was used to align the reads to the human reference genome hg19, and duplications were removed according to the previous study [19, 20]. The subsequent analysis was performed by an in-house developed bioinformatic pipeline. The reads were annotated through the latest ANNOVAR in hg19 coordinates [21]. The list of genomic regional elements was from the UCSC genome browser [22] and previous study [23–25].

## 3. Results

### 3.1. Cell-Free DNA Fragment Size Distribution and Concentration in the Plasma

**Figure 1:**
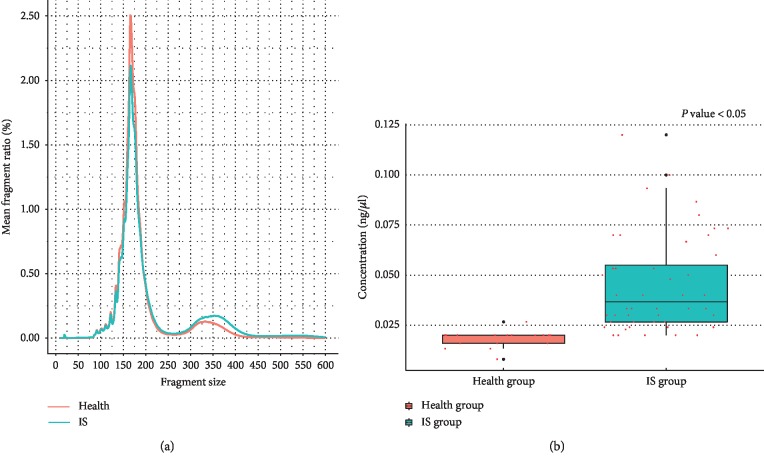
The length distribution and the concentration of cell-free DNA. (a) The mean fragment ratio of specific fragment size in the two groups; (b) the concentration difference of plasma cell-free DNA in the two groups.

The length distribution of plasma cell-free DNA fragments revealed the average fragment ratio of specific fragment size in two groups. It showed that the proportion of plasma cell-free DNA fragments (75–250 bp) in the healthy group was significantly greater than that of the IS patients (Figure 1(a)). However, the fragment (300–400 bp) proportion of the IS group is significantly greater than that of the healthy group (Figure 1(a)). The results of cell-free DNA concentration in the plasma showed that the IS group is also greater than that of the healthy group (Figure 1(b), *P* < 0.05).

### 3.2. Distribution of Cell-Free DNA in Gene Components

**Figure 2:**
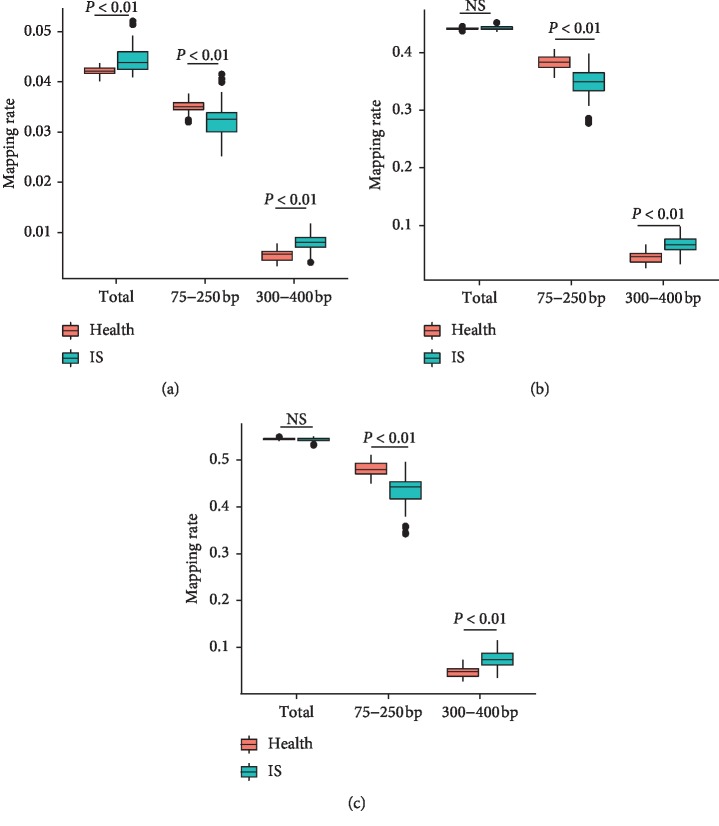
The distribution of cell-free DNA on the genetic element. The ratio of fragments in each genome element was counted. Orange bar represents the ratio of fragments in the healthy group. Green bar represents the ratio of fragments in IS samples. *P* values were calculated by Student's *t*-test. (a) In EXON region, (b) in INTRON region, and (c) in INTERGENIC region.

Overall speaking, cell-free DNA in both the IS group and the healthy group was evenly distributed across different chromosomes, despite the two groups' data exhibited some differences ([Supplementary-material supplementary-material-1]). The distribution of plasma cell-free DNA (total fragments) in the INTRON and INTERGENIC regions showed no significant difference between the two populations (Figure 2). However, the IS group has higher ratio of fragments in the EXON region (Figure 2). Meanwhile, there were significant differences in the distribution of different fragment lengths. In general, the fragment proportion of the healthy group is higher in the range of 75–250 bp, and the fragment proportion of IS patients is higher in the 300–400 region (Figure 2).

### 3.3. Distribution of Plasma Cell-Free DNA in Genomic Components

**Figure 3:**
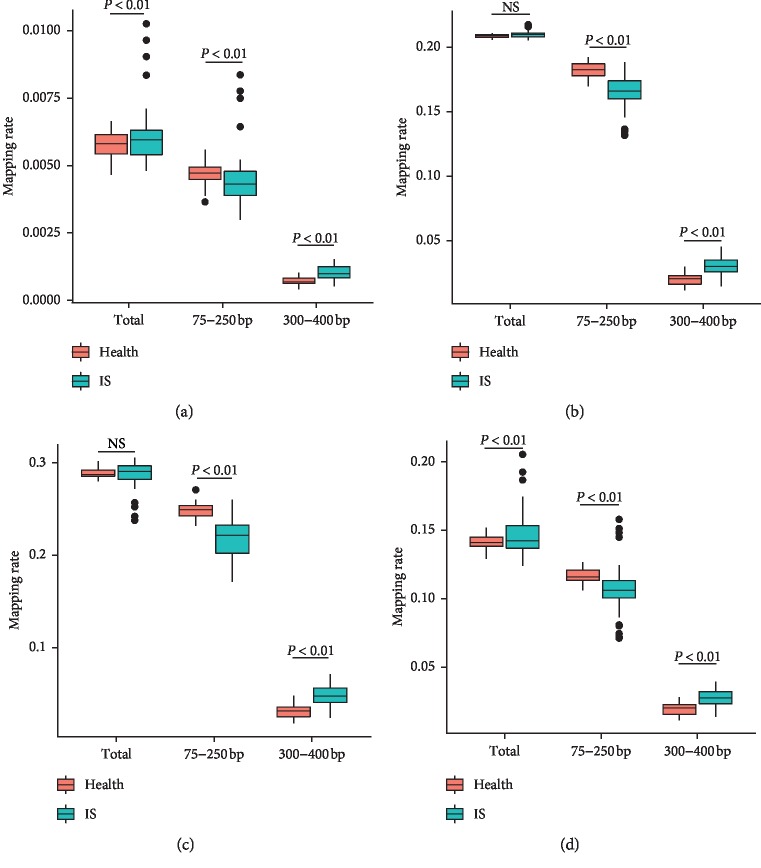
The distribution of cell-free DNA on the gene element. The ratio of fragments in each genome element was counted. Orange bar represents the ratio of fragments in the healthy group. Green bar represents the ratio of fragments in the IS group. *P* values were calculated by Student's *t*-test. (a) In CpG islands, (b) in Frag region, (c) in LINE region, and (d) in ALU region.

The results showed that fragments (total fragments) of the IS group were strongly enriched in the regions of Alu and CpG islands, in contrast to the data of the healthy group. In the Fragile and LINE regions, the fragment ratio of the IS group showed no obvious difference between the IS group and the healthy group (Figure 3). Among these genomic components, the healthy group displayed a greater ratio of fragments (75–250 bp) compared to the IS patient population; whereas, the fragment (300–400 bp) proportion in the IS group was significantly greater than that of the healthy group (Figure 3).

## 4. Discussion

The distribution of plasma cell-free DNA fragment length is known to be closely related to the multiple disease state. However, the distribution of fragment length in stroke patients is still unclear. Recently, researchers had revealed that the distribution of nucleosome spacing displayed regularity in the healthy group, whereas aberrant nucleosome spacing could be detected in many diseases [26–30]. Based on this fact, our speculation for stroke is as follows: at the early stage of stroke, cell death programs (apoptosis, acute necrosis, chronic inflammation, etc.) may be resulting a distinctive nucleosome spacing profiles of plasma cell-free DNA. The plasma cell-free DNA content, length, distribution of fragments on different genetic components, etc., could change regularly, which may be correlated with and reflecting the state, severity, and prognosis of stroke cases. As detected in all the samples, the plasma cell-free DNA mainly peaked at ∼170 bp and ∼350 bp, despite that a greater ∼350 bp peak and a smaller ∼170 bp peak were detected in the stroke group as compared to the respective peaks in the healthy group. It has been known that the approximate size of the mono-nucleosomal DNA fragments released is 180 bp. Our study revealed that there are multiple relationships between the fragment sizes of two main peaks in the plasma samples; therefore, it might suggest that this cell-free DNA comes from apoptotic cells [31–33]. The proportion differences in length might be indicating that the fragmentation process of plasma cell-free DNA was altered in stroke, but the scenario remained unknown.

The overall chromosomal distribution of the plasma cell-free DNA indicated that multiple regions have different fragment ratios between IS patients and the health group across the whole genome. Therefore, we analyzed the distribution of plasma cell-free DNA on various genetic components in this study. It was intriguing to note in the in-depth analysis that the general distribution of plasma cell-free DNA in the stroke group was more prone to the regions of ALU, CpG islands, and EXON when compared to the healthy group. This phenomenon might indicate that the DNA degradation in the two groups of samples has different inclination in these regions. Moreover, the distribution patterns in the 75–250 bp and 300–400 bp ranges displayed differences. In conclusion, the genomic regional distribution of plasma cell-free DNA fragment data identified major differences between the stroke group and the healthy group, providing a theoretical basis of deploying plasma cell-free DNA fragmentation data to assess stroke risks and a clinical application possibility in the near future.

## Data Availability

The sequencing data used to support the findings of this study are restricted by the Ethics Review Committee of Jining Medical University in order to protect the patient's privacy. Data are available from the corresponding author for researchers who meet the criteria for access to confidential data.

## Supplementary Materials

Supplementary Materials Table S1: the data production of high-throughput sequencing. Figure S1: the distribution of cell-free DNA on the chromosome.
