# Supplementary figures and images for: The Length and Distribution of Plasma Cell-Free DNA Fragments in Stroke Patients
Source: Biomed Res Int. 2020 Jan 30;2020:9054196. doi: 10.1155/2020/9054196 (PMC7017581; doi:10.1155/2020/9054196)

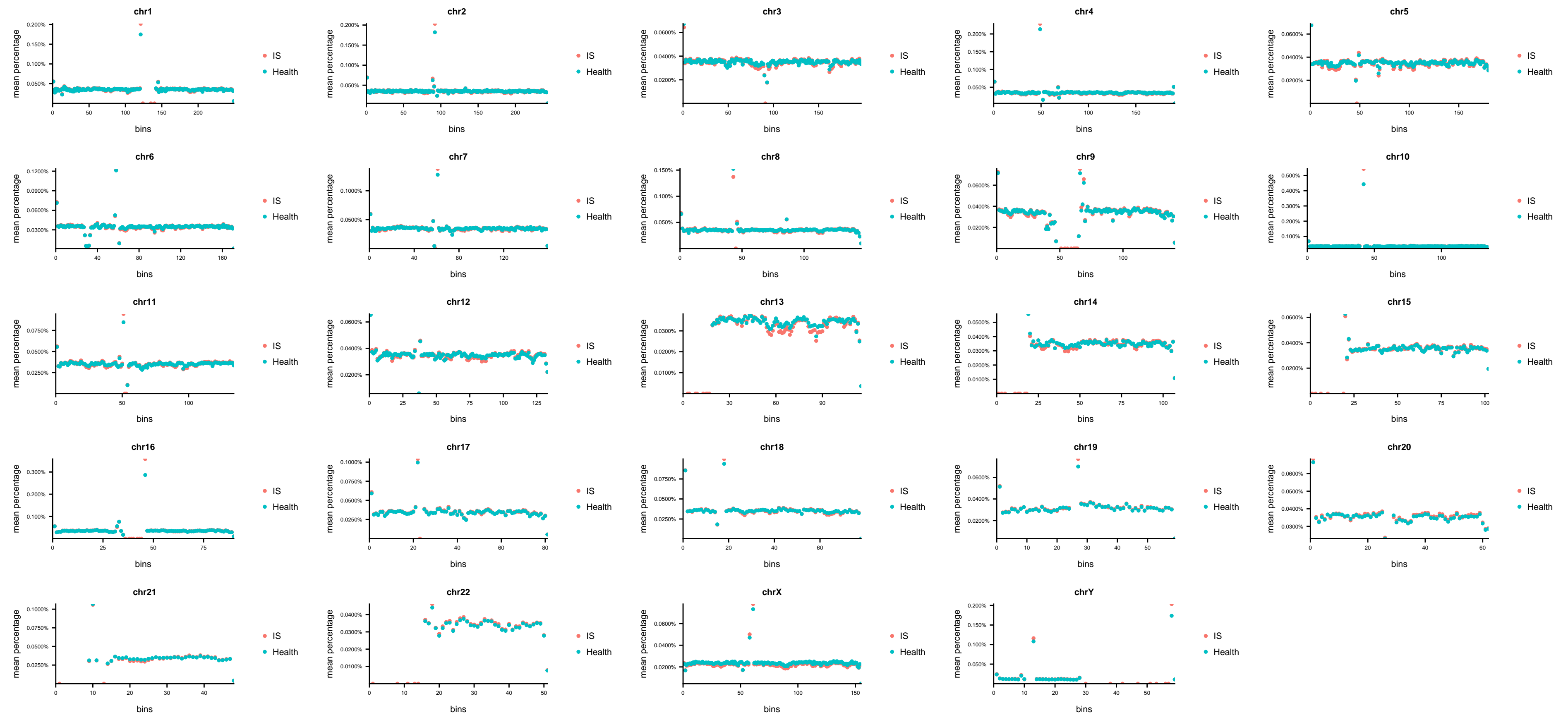

Supplement: Supplementary Materials — Table S1: the data production of high-throughput sequencing. Figure S1: the distribution of cell-free DNA on the chromosome. [file 9054196.f1.zip › 9054196.f1/FigS1.pdf]
